# Mitochondrial Genome Analysis of Wild Rice (*Oryza minuta*) and Its Comparison with Other Related Species

**DOI:** 10.1371/journal.pone.0152937

**Published:** 2016-04-05

**Authors:** Sajjad Asaf, Abdul Latif Khan, Abdur Rahim Khan, Muhammad Waqas, Sang-Mo Kang, Muhammad Aaqil Khan, Raheem Shahzad, Chang-Woo Seo, Jae-Ho Shin, In-Jung Lee

**Affiliations:** 1 School of Applied Biosciences, Kyungpook National University, Daegu, 41566, Republic of Korea; 2 Chair of Oman's Medicinal Plants & Marine Natural Products, University of Nizwa, Nizwa, 616, Oman; 3 Department of Agriculture, Abdul Wali Khan University Mardan, Mardan, Pakistan; Institute of Crop Sciences, CHINA

## Abstract

*Oryza minuta* (Poaceae family) is a tetraploid wild relative of cultivated rice with a BBCC genome. *O*. *minuta* has the potential to resist against various pathogenic diseases such as bacterial blight (BB), white backed planthopper (WBPH) and brown plant hopper (BPH). Here, we sequenced and annotated the complete mitochondrial genome of *O*. *minuta*. The mtDNA genome is 515,022 bp, containing 60 protein coding genes, 31 tRNA genes and two rRNA genes. The mitochondrial genome organization and the gene content at the nucleotide level are highly similar (89%) to that of *O*. *rufipogon*. Comparison with other related species revealed that most of the genes with known function are conserved among the Poaceae members. Similarly, *O*. *minuta* mt genome shared 24 protein-coding genes, 15 tRNA genes and 1 ribosomal RNA gene with other rice species (*indica* and *japonica*). The evolutionary relationship and phylogenetic analysis revealed that *O*. *minuta* is more closely related to *O*. *rufipogon* than to any other related species. Such studies are essential to understand the evolutionary divergence among species and analyze common gene pools to combat risks in the current scenario of a changing environment.

## Introduction

In recent years, we have noticed a significant increase in the sequencing of organelle genomes, particularly those of economically important crop plants. To date, 300 mitochondrial (mt) and 342 complete chloroplast (cp) genomes have been submitted to GenBank Organelle Genome Resources. Compared to fungi and animal mitochondrial genomes, plant mitochondrial genomes are more complex and encode a higher number of genes. These genomes play vital roles in plant development and productivity [1–3]. There is an unusual size variation found in seed plant mt genomes, especially within the same family [4]. Seed plant mt genomes are distinctive for their frequent insertion of foreign DNA by gene transfer [5], very low mutation rate [6] and dynamic structure [7]. Terrestrial seed plants have increased their mt genome sizes by adopting new mechanisms to facilitate more gene exchange between nuclear genomes and mt genomes as well as cp genomes and mt genomes [8]. Investigations of the mt genomes of different important angiosperm species, including *Beta vulgaris* [9], *Arabidopsis thaliana* [10], *Brassica napus* [11, 12], *Triticum aestivum* [1], *Oryza sativa* [13, 14], *Zea mays* [15–17], *Vitis vinifera* [18], *Nicotiana tabacum* [19], *Vigna radiata* [20] and *Citrullus lanatus* [4], have been performed. Together with physical mapping [21–23], these mt genomes showed various properties, such as a slow rate of evolution, large genome size (200–2400 kb), multipartite structure, uptake of foreign DNA and different modes of gene expression (RNA editing and splicing) [24]. However, the above properties are unable to explain the diversity of mitotypes within each plant genus and species.

Much of the structure and size difference in plant mt genomes are repeated sequences in the DNA content [25]. The repeated DNA sequences are also sources for intragenomic recombination, and they trigger various changes in mitochondrial genome evolution and structural dynamism [26]. To analyze the evolutionary distinctiveness of a plant’s mitochondrial genome within one plant genus or species, more systematic and specific sequences are needed. To date, there are no specific and organized sequences for an angiosperm genus to analyze multiple species for mitochondrial genome derivation; therefore, the mechanism of having such uniqueness has not yet been revealed [26].

Previously, phylogenetic analysis [27–29] has reported the complicated and laborious method of amplifying selected loci in genes, some of which are unable to provide sufficient and accurate information about phylogenetic resolutions. Recently, next generation sequencing advancements have led to various organelle genome sequencing, which is continuously contributing to various areas of biology. The use of whole organelle genome sequencing, especially chloroplast and mitochondria genomes, has been recently demonstrated as a potential barcode [30] that can assist in overcoming the previous process of collecting data over generations. Furthermore, due to recombination in the nucleus, data may lead to unreliable phylogenies; organelles are structurally stable, non-recombinant, haploid and have certain advantages in phylogenetic reconstructions [31].

Rice is one of the most important cereal crops, a staple food for more than half of the world’s population and a model crop for cereal genomics. The genus *Oryza* has two cultivated species and more than 20 wild relatives based on pairs of chromosomes. *Oryza* species are categorized into 10 genome types: AA, BB, CC, BBCC, EE, FF, GG, CCDD, HHJJ and HHKK [27]. Furthermore, these genome types have different species and subspecies. *O*. *sativa*, one of the important species that has an AA genome type, is further divided into the following subspecies: *O*. *sativa* L. spp. *japonica* and *O*. *sativa* L. spp. *indica*, which has a global distribution [32]. Moreover, wild *Oryza* species have the potential to resist against biotic and abiotic stresses, especially to insect pests (Heinrichs et al., 1985). *O*. *minuta*, a tetraploid wild relative of cultivated rice with a BBCC genome, exhibits the potential to resist against blast blight, bacterial blight (BB), white backed planthopper (WBPH) and brown plant hopper (BPH) diseases. Furthermore, various resistance genes have been transferred successfully to cultivated rice from *O*. *minuta* [33, 34]. These wild and cultivated species share a valuable genetic diversity that has contributed greatly to the improvement of rice crops. To identify more desired genes and ensure effective conservation, analysis of their phylogenetic and evolutionary relationship is very important [35]. Hence, the current study was performed. Various organelle genomes of Poaceae members have already been reported, including *O*. *sativa indica*, *O*. *rufipogon*, *O*. *sativa japonica*, *T*. *aestivum* and *Z*. *mays* [13, 36–38]. Recently, many nuclear genomes from various economically important plants have been published or are still in progress [39]. Billions of short read sequencing data for the whole genome from many species are deposited in a public database. In this study, we aimed to analyze the complete mitochondrial genome sequence of *O*. *minuta* (mtDNA) and compare it with other sequenced mt genomes of the Poaceae family. The current study will provide information for the further understanding of mt genome evolution in related species.

## Materials and Methods

In this study, we successfully assembled and annotated the complete mtDNA of a wild cultivar of *O*. *sativa* (IRGC 101140) following the method described previously [40, 41]. Approximately sixty million raw Illumina reads were demultiplexed and trimmed. The raw reads were filtered and then assembled *de novo* into contigs using CLC Genomics Workbench v7.0 (CLC Bio, Aarhus, Denmark).

### Sequence data analysis

BLAST searches were conducted on all of the contigs using the NCBI database (http://www.ncbi.nlm.nih.gov/) for the annotation of mitochondrial sequences using previous angiosperm annotated mitochondrial genes as query sequences. tRNA scan-SE software (http://lowelab.ucsc.edu/tRNAscan-SE/) was used to identify tRNAs in the genome. The ORF-Finder (http://www.ncbi.nlm.nih.gov/gorf/gorf.html) was used to predict and annotate open reading frames (ORFs) with a minimum size of 100 codons. Analysis of repeat sequences was performed as described previously [42]. While the circular map of mt genome was created using OGDraw v1.2 (http://ogdraw.mpimp-golm.mpg.de/), the tandem repeats were identified with Tandem Repeat Finder (TRF) using a default setting [43]. The tandem repeat lengths were set to 20 bp or more with a maximum period size and a minimum alignment score of 500 and 50, respectively, and the repeats identity was set to >80%. The annotated genome sequence was submitted to NCBI with the GenBank accession No. KU176938.

### Comparing mitochondrial genomes and evolutionary analysis

The *O*. *minuta* mitochondrial genome (GenBank: KU176938) sequence described here was compared with seven other reported Poaceae mitotypes: *O*. *sativa japonica* (GenBank: BA000029), *O*. *sativa indica* (GenBank: DQ167399), *O*. *rufipogon* (GenBank: AP011076), *Triticum aestivum* (GenBank: NC007579), *Zea mays* spp. *parviglumis* (GenBank: NC008332), *Z*. *mays* spp. *mays* (GenBank: NC007982) and *Sorghum bicolor* (GenBank: NC008360), using NCBI-blastn. For comparison, 20 protein coding genes (*atp9*, *ccmC*, *ccmFN1*, *cox1*, *cox2*, *cox3*, *cob*, *matR*, *nad4L*, *nad6*, *nad9*, *rps1*, *rps3*, *rps7*, *rps12*, *rps13*, *rps4*, *rrn5*, *rpl2*, and *rpl5*), which were shared by these eight species, were extracted and successively joined together. MEGA 6 [44] was used to construct a neighbor-joining tree [45] with 1000 bootstrap replications [46]. For the whole genome as well as the coding regions, comparison distance matrices were computed using Progressive Mauve (The Darling lab at the University of Technology Sydney), and then the whole genome distance matrix was converted to a heat map [47]. Furthermore, the dot matrix method was also used to analyze similarities among different sequences [48].

## Results

### Mitochondrial genome of *O*. *minuta*

Mitochondrial DNA of *O*. *minuta* was assembled into a circular genome of 515,022 bp with 44% overall GC content, which is almost similar to the mtDNA of other related species (Table 1). The non-coding sequences of *O*. *minuta* mtDNA is almost 86.04%, which is less than the previously reported angiosperm average non-coding sequences content (89.46%) [29]. Genes account for 13.9% of the genome and 71,846 bp in length.

**Table 1:** Gene contents and total length of *Oryza* species mitogenomes.

Features	*O*. *minuta*	*O*. *sativa j*	*O*. *sativa i*	*O*. *rufipogon*
**Genome size (bp)**	515,022	490,520	491,515	559,045
**GC contents**	44	43.9	43.8	44
**Total gene contents**	93	81	94	59
**Protein coding gene**	60	56	53	41
**rRNA**	2	3	6	3
**tRNA**	31	22	33	15
**Total gene length**	71,846	53,182	156,514	43,715

### Gene content and open reading frames (ORFs)

**Fig 1:**
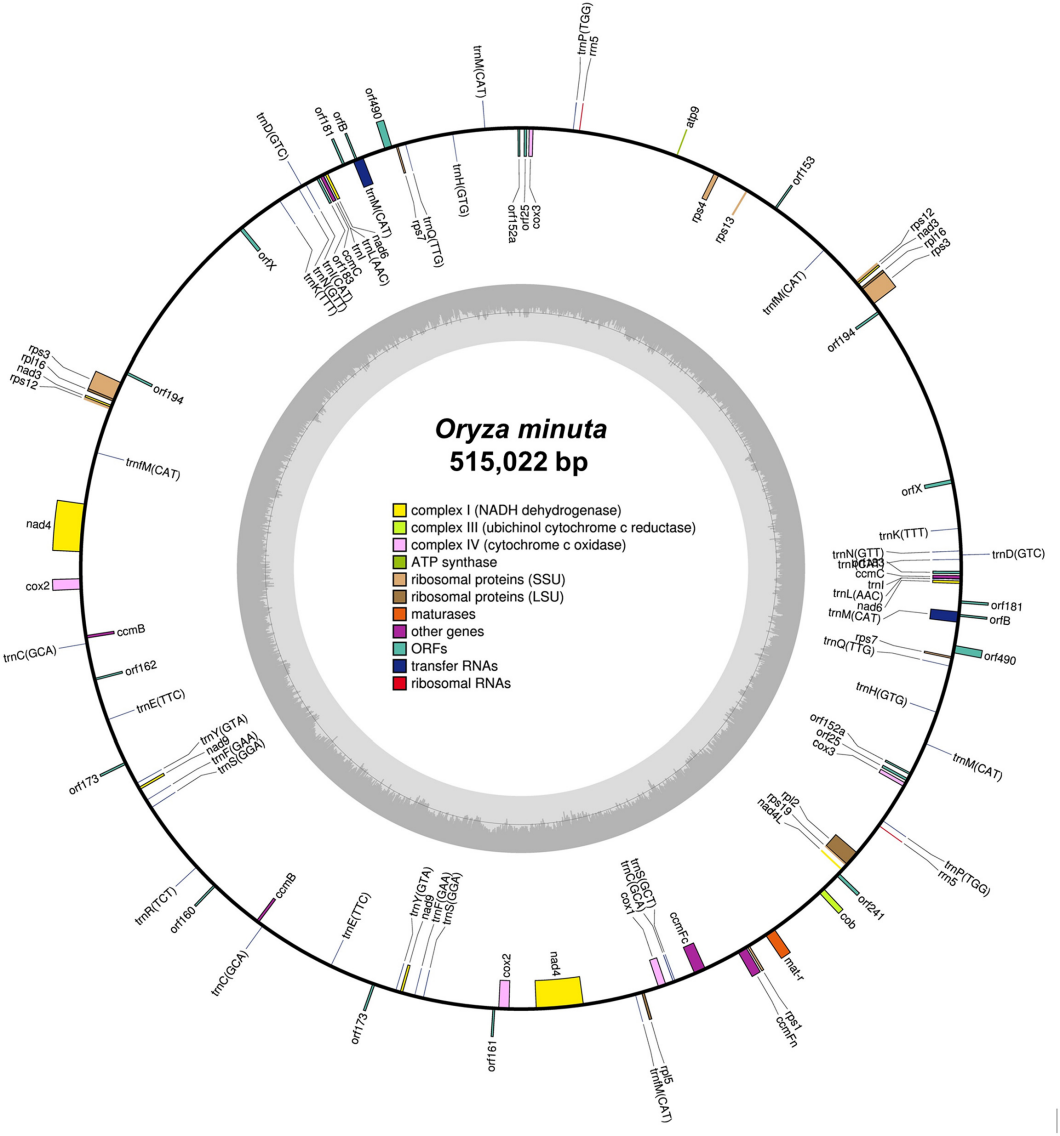
Mitochondria genome map of *O*. *minuta*. Features on the clockwise- and counter-clockwise transcribed strands are drawn on the inside and outside of the circle, respectively.

A total of 93 genes were identified, including 60 protein-coding genes (PCGs), 31 tRNA genes and 2 rRNA genes using BLAST and TRNA scane-SE (Fig 1 and Table 1). The protein coding genes were present in a range of 225 bp (*atp9*) to 8,980 bp (*nad4*), which included 31 genes for the production of ATP synthase and the electron transport chain, consisting of the following subunits: 4 subunits of complex I (*nad3*, *4*, *6*, *9*), 1 subunit of complex III (*cob*), 3 subunits of complex IV (*cox1-3*) and 1 subunit of complex V (*atp9*) (Table 2). Furthermore, there were four genes for cytochrome c biogenesis (*ccmB*, *ccmC*, *ccmFN* and *ccmFC*), three genes for large ribosomal proteins (*rpl2*, *5*, *16*) and seven genes for small ribosomal protein (*rps1*, *3*, *4*, *7*, *12*, *13*, and *19*) (Table 2). The total length of the 60 protein coding genes of *O*. *minuta* mtDNA was 71, 846 bp (Table 1), accounting for 13.9% of its total genome length, which is different from other *Oryza* genus mitogenomes. In *O*. *minuta*, the most common start codon for the protein coding genes was ATG, except for *ccmB* (start codon ATC), *matR* (start codon AGA) and *rrn5* (start codon AAA), as reported previously (Handa, 2003). Ten genes (*ccmB*, *cox3*/*3*, *orf160*, *orf194*, *orf241*, *rps1*, *rps12*/*12* and *rps13*) were expected to terminate with TGA and eleven (*ccmC*, *ccmFn*, *cob*, *cox1*, *cox2*, *mat-R orf25/orf153*, *orf194*, *orfx*, *rps3*) with TAG; other protein coding genes use TAA as their termination codon.

**Table 2:** Gene contents comparison of *O*. *minuta* mitochondria genome with other *Oryza* species.

Product group	Gene	O.m	O.s. i	O. s. j	O.r	Product group	Gene	O.m	O.s. i	O. s. j	O.r
**Complex I**	*nad3*	+2	+	+	+	**Cytochrome C**	*ccmB*	+	+	+	-
	*nad4*	+2	+	+	+		*ccmC*	+	+	+	+
	*nad6*	+2	+	+	+		*ccmFc*	+	+	+	-
	*nad9*	+2	+	+	+		*ccmFn*	+	+	+	+
**Complex III**	*cob*	+	+	+	+	**Intron maturase**	*mat-r*	+	+	+	+
**Complex IV**	*cox1*	+	+	+	+	**tRNA**					
	*cox2*	+	+	+	+	**Cysteine**	*trnC*	+2	+2	+	+
	*cox3*	+	+	+	+	**Aspartic**	*trnD*	+2	+	+	+
**Complex V**	*atp9*	+	+	+	+	**Glutamic**	*trnE*	+2	+2	+	+
**Ribosomal large subunit**	*rpl2*	+	+	+	+	**Phenylalanine**	*trnF*	+2	+2	+	+
	*rpl5*	+	+	+	+	**Methionine**	*trnM*	+2	+3	+2	+2
	*rpl16*	+	+	+	+	**Histidine**	*trnH*	+2	+2	+	+
**Ribosomal small subunit**	*rps1*	+	+	+	+	**Isoleucine**	*trnl*	+2	+2	+	+
	*rps3*	+	+	+	+	**Lysine**	*trnK*	+2	+	+	+
	*rps4*	+	+	+	+	**Leucine**	*trnL*	-	+3	+	-
	*rps7*	+	+	+	+	**Asparagine**	*trnN*	+2	+1	+	-
	*rps12*	+	+	+	+	**Proline**	*trnP*	+2	+4	+	+
	*rps13*	+	+	+	+	**Glutamine**	*trnQ*	+2	+2	+	+
	*rps19*	+	+	+	+	**Arginine**	*trnR*	+1	+1	+	+
**rRNA**						**Serine**	*trnS*	+3	+4	+3	+3
**rRNA genes**	*rrn5*	+2	+	+	+	**Tyrosine**	*trnY*	+2	+2	+	+

O.m = *O*. *minuta*, O.s. i = *O*. *sativa indica*, O. s. j = *O*. *sativa japonica*, O.r = *O*. *rufipogon*.

A total of 31 tRNA sequences (2,328 bp) were found in O. minuta mtDNA (Table 1) in the range of 71–88 bp in length. The GC content of the tRNA genes was 51.3%, with A, C, G, and T as 22.1, 22.6, 28.7 and 26.6%, respectively, which was higher than the overall GC composition of the mtDNA. Among these genes, tRNAs for 16 amino acids, including seven for Methionine (Met), three for serine (Ser), two for Lysine (Lys) and Cysteine (Cys), are encoded (Table 3). The genome deficient tRNAs for the following amino acids were: Valine (Val), Alanine (Ala), Leucine (Leu), Glycine (Gly), Tryptophan (Trp) and Threonine (Thr) (Table 3). A total of 627 ORFs were identified, which was longer than 100 codons in the *O*. *minuta* mitochondrial genome using ORF-Finder. All ORFs were a single copy between 200 and 800 bp in length, except for nine ORFs that were longer than 2,000 bp, including the 2,508 bp *orf492* and the 3,474 bp *orf5*.

**Table 3:** Recognition of anticodons by tRNA genes found in the mitochondrial genome of *O*. *minuta*.

NAME	Type	Anticodon	Length(bp)	Orientation
trnQ	Gln	(TTG)	72	Inverted
trnH	His	(GTG)	74	Inverted
trnM	Met	(CAT)	73	Direct
trnP	Pro	(TGG)	75	Direct
trnS	Ser	(GCT)	88	Inverted
trnfM)	Met	(CAT	74	Direct
trnS	Ser	(GGA)	87	Inverted
trnF	Phe	(GAA)	73	Inverted
trnY	Tyr	(GTA)	83	Inverted
trnE	Glu	(TTC)	72	Inverted
trnC	Cys	(GCA)	71	Direct
trnR	Arg	(TCT)	72	Direct
trnS	Ser	(GGA)	87	Inverted
trnF	Phe	(GAA)	73	Inverted
trnY	Tyr	(GTA)	83	Inverted
trnE	Glu	(TTC)	72	Inverted
trnC	Cys	(GCA)	71	Direct
trnfM	Met	(CAT)	74	Inverted
trnK	Lys	(TTT)	73	Inverted
trnN	Asn	(GTT)	72	Inverted
trnD	Asp	(GTC)	74	Direct
trnI	Ile	(CAT)	74	Inverted
trnQ	Gln	(TTG)	72	Inverted
trnH	His	(GTG)	74	Inverted
trnM	Met	(CAT)	73	Direct
trnP	Pro	(TGG)	75	Direct
trnfM	Met	(CAT)	74	Inverted
trnK	Lys	(TTT)	73	Inverted
trnN	Asn	(GTT)	72	Inverted
trnD	Asp	(GTC)	74	Direct
trnI	Ile	(CAT)	74	Inverted

### Repetitive sequences in the mitochondrial genome

Large repeats were identified in the mitochondrial genome of *O*. *minuta*. Seven pairs of repetitive sequences were found and designated as R1-R7 (Table 4). R1-R4 (19,773, 16,451, 7,984, 6,856 bp) had pairs of large repeats in the same direction longer than 6,050 bp, while R5-R7 (112, 82, 70 bp) had inverted repeats longer than 70 bp. The large repeat from R2 contained two genes, *trnE* and *orf173*, while R3 contained the *trnK* gene. No protein-coding gene was found in the other repeats. Furthermore, a total of 22 tandem repeats of more than 10 bp were identified in the *O*. *minuta* mitogenome (Table 5). The length of the repeat units in these regions varied between 11 and 70 bp, and up to 3 repeat units presented with having a varied identity percentage from 80 to 100% (Table 5).

**Table 4:** Large repeats in the mitochondrial genome of *O*. *minuta*.

No	Type	Size	Copy-1	Copy-2	Difference between copies	Identity
R1	DR	19773	20563–40336	189513–209286	identical	100
R2	DR	16451	279979–296430	343373–359824	identical	100
R3	DR	7984	3630–11614	172517–180501	identical	100
R4	DR	6856	55990–62846	225087–231943	identical	100
R5	IR	112	408736–408848	235292–235374	identical	100
R6	IR	82	57033–57124	66037–66126	2bp	99%
R7	IR	70	47047–47116	51677–51746	identical	100%

**Table 5:** Distribution of tandem repeats in the *O*. *minuta* mitochondrial DNA.

S/No	Indices	Repeat length	*Size of repeat ᵡ Copy Number*	Percent Matches
1	169–216	19	47.5	89
2	27345–27372	11	27.5	100
3	39540–39584	23	46	91
4	99616–99653	17	34	95
5	115088–115120	16	32	94
6	137893–137929	18	36	100
7	169070–169117	19	38	89
8	187893–187937	23	46	90
9	187891–187942	23	46	80
10	196295–196322	11	22	100
11	208490–208534	23	46	91
12	256219–256251	15	30	100
13	271765–271790	13	26	100
14	302563–302704	70	140	100
15	312455–312504	25	50	100
16	334733–334782	25	50	100
17	387201–387233	15	33	100
18	412175–412207	15	33	100
19	415961–416025	32	80	86
20	458189–458229	21	42	90
21	484076–484112	18	36	100
22	496080–496179	52	104	95

### *O*. *minuta* mtDNA comparison with other *Oryza* species

We compared the sequences of the mtDNA from *O*. *minuta* (515,022 bp) with three *Oryza* species: *O*. *sativa indica*, *O*. *sativa japonica* and *O*. *rufipogon*, which all have circular mitochondrial genomes of 491,515, 490,520 and 559,045 bp, respectively (Table 1). The mitochondrial genome of *O*. *minuta* was larger than *O*. *sativa indica* and *O*. *sativa japonica*, while smaller than *O*. *rufipogon* (Table 1). The GC content of *O*. *minuta* was slightly different from other mitogenomes. Similarly, nucleotide base content of the total length of the genes with known functions (71,846 bp) was different among these mitogenomes (Table 1). Analysis of the genes with known functions showed that *O*. *minuta* shared 24 protein encoding genes, 15 tRNA genes and 1 ribosome gene (Table 2); paralogous genes that presented in more than one copy were counted here as one gene. The numbers of genes with known functions were almost the same in these mitogenomes, but the total number of genes varied, ranging from 59 in *O*. *rufipogon* to 94 in *O*. *sativa indica* (Table 1).

### Evolutionary relationships of the *O*. *minuta* mitogenome

To explain the evolutionary relationship of *O*. *minuta* within the Poaceae family, the mtDNA genomes of selected species were compared with related mtDNA sequences using blastn. Similar regions in these mitogenomes were aligned to the mtDNA of *O*. *minuta* (reference genome). The *O*. *minuta* sequence showed 89% identity to that of the *O*. *rufipogon* mtDNA sequences. The sequence identity shared by the mtDNA of *O*. *minuta* with *O*. *sativa* (*indica* and *japonica*), *S*. *bicolor*, *T*. *aestivum* and *Z*. *mays* (*mays* and *parviglumis*) were 34.2, 34.2, 10.1, 17.8, 14.1 and 13.3%, respectively (S1 Fig). These results strongly suggested that *O*. *minuta* was closely related to *O*. *rufipogon*, and the evolutionary relationship between these two was much stronger than that of *O*. *minuta* with any other species.

**Fig 2:**
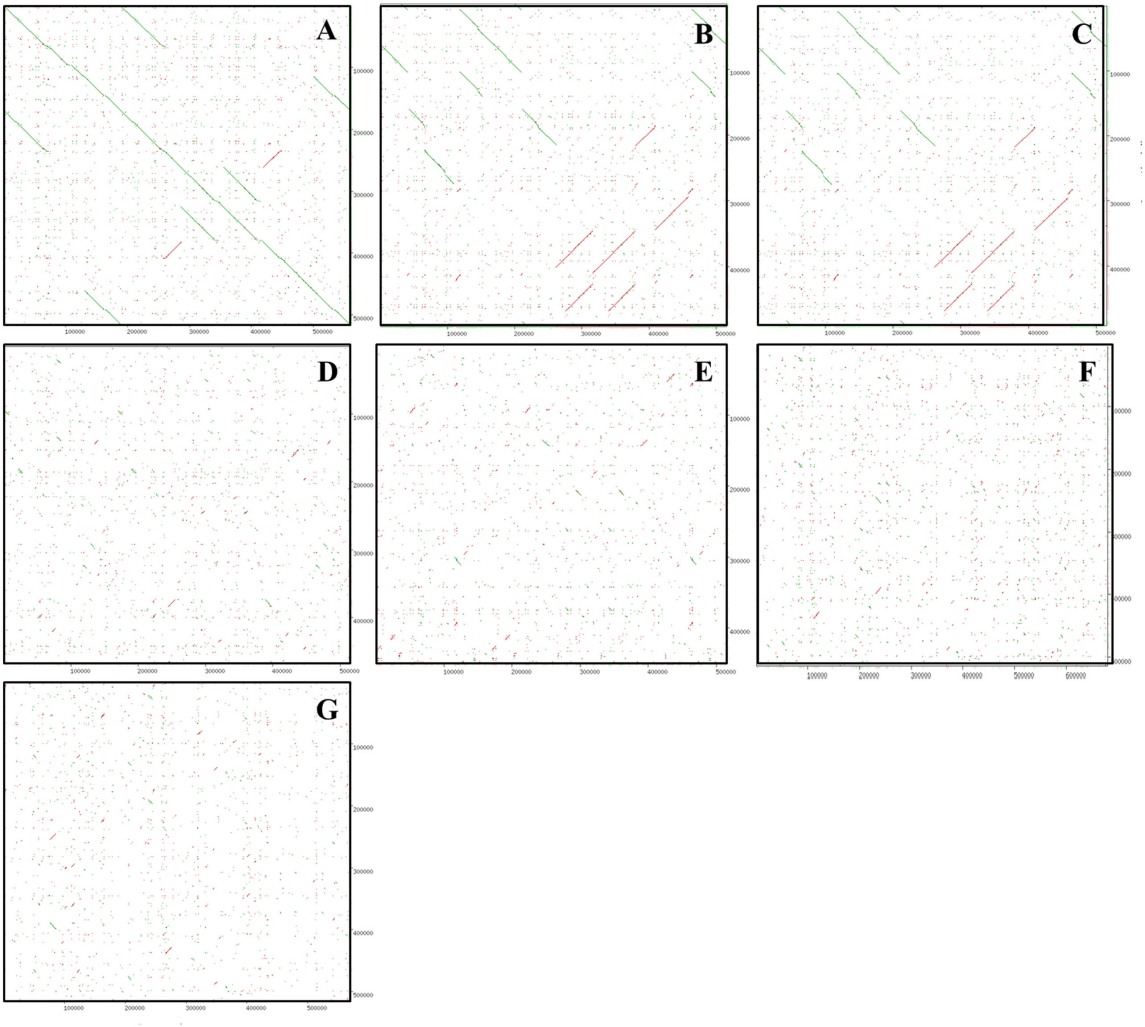
Dot matrix alignment of the *O*. *minuta* (x-axis) with other mitochondrial genomes of Poaceae members (y-axis). (A) *O*. *rufipogon*, (B) *O*. *sativa japonica*, (C) *O*. *sativa indica*, (D) *S*. *bicolor*, (E) *T*. *aestivum*, (F) *Z*. *mays* spp. *parviglumis* and (G) *Z*. *mays* spp. *mays*.

**Fig 3:**
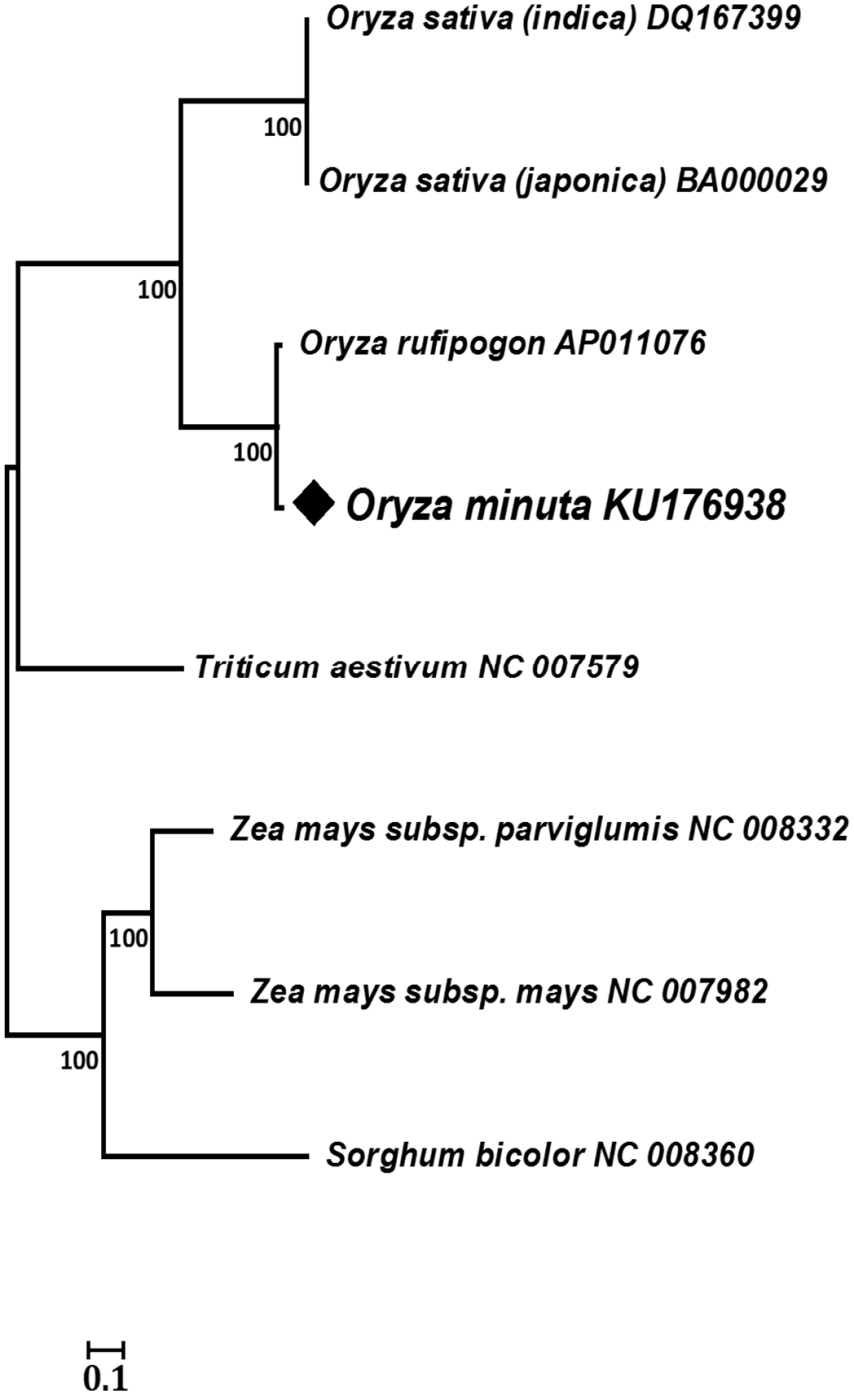
Phylogeny of the *O*. *minuta* mitogenome with seven other Poaceae members. The phylogenetic tree was inferred using the neighbor-joining method based on 20 conserved genes.

To support these results, a dot matrix analysis showed that the length of syntenic regions of *O*. *minuta* with the *O*. *rufipogon* mitogenome were longer and straight. Additionally, the identity of *O*. *minuta* with *O*. *sativa indica* and *japonica* was lower, and the distribution of the syntenic regions was more dispersed than that of *O*. *rufipogon* (Fig 2A–2C). Moreover, the phylogenetic relationships among the Poaceae members (Fig 3; S2 Fig) were conducted using 20 conserved genes among the reported mitogenomes by the neighbor-joining method. These results were consistent with our comparative results based on mitochondrial genome analysis and revealed that *O*. *minuta* was more closely related to *O*. *rufipogon* than any other Poaceae member.

## Discussion

The Poaceae is an important plant family with significant importance to human beings because the plants in this family are the main sources for human food and animal feed. The rapid increases in genomic analysis and bioinformatics approaches have revealed the great agronomic and scientific importance of this model species. Furthermore, mitochondrial genome analysis of closely related species has significantly improved the knowledge of molecular evolution and phylogenetic analysis [49] in various species. *O*. *minuta*, a wild rice with the BBCC genome, has been used as a donor of resistance to bacterial diseases. Because of its important agronomic traits and characteristics, this species is very important for rice breeders [50]. To further understand this important species, its mitochondrial genome was sequenced, annotated and compared with other related species.

The mitochondrial genomes of Poaceae members were generally larger in size (452.52–704 kb) compared with other angiosperm plants. The *O*. *minuta* mtDNA (515 kb) was larger than *O*. *sativa* (*indica* and *japonica*) while smaller than *O*. *rufipogon* (559.04 kb). Similarly, the percentage of the GC contents were slightly different from *O*. *sativa* (*indica* and *japonica*) (43.8 and 43.9) and similar with *O*. *rufipogon* [8, 13, 36]. Comparison of the *O*. *minuta* mtDNA with the three mitogenomes above revealed that the protein coding genes were relatively conserved among these mitogenomes. A total of 24 coding genes, 15 tRNA genes and 1 ribosomal rRNA gene were shared within these mitogenomes. However, various genes (*ccmB*, *ccmC*, *cox2*, *cox3*, *nad3*, *nad4*, *rpl16*, *rps12*, *rps3*, *rps7* and *rrn5*) were present in the duplication of the *O*. *minuta* genome. Furthermore, genes (*ccmB*, *ccFc*, *nad4*, and *rpl16*) were absent in the *O*. *rufipogon* mitogenome [8] (Table 2).

A total of 31 tRNA sequences for 15 genes were identified in *O*. *minuta* mtDNA, accounting for only 0.40% of the mitochondrial genome (Table 1). Among them, six genes exhibited high sequence similarity (>99%) to the chloroplast genome and seemed to be derived from the chloroplast. The chloroplast-derived genes (*trnW-CCA*, *trnD-GUC*, *trnS-GGA*, *trnH-GTG*, *trnM-CAT* and *trnL-CAA)*, which are commonly found in angiosperm mitochondrial genomes [51], were present in the *O*. *minuta* mitogenome. Furthermore, another two genes, *trnQ-UUG* and *trnP-GGG* reported in dicot transfer events [52, 53], were additionally found in *O*. *minuta*. Thus, functional tRNA for eight amino acids (*trnB*, *trnA*, *trnT*, *trnV*, *trnZ*, *trnW and trnX*) were absent from the *O*. *minuta* mitogenome, although for protein synthesis in the mitochondria, tRNAs for 20 amino acids are necessary. These results revealed that the nuclear genome might have supplied these missing tRNAs. Thus, nine tRNAs involved in mitochondria biogenesis in rice are of mitochondrial origin, six are of plastid origin and the above missing are probably of nuclear origin. These results paralleled results previously reported for the *O*. *sativa* mitogenome [13]. Furthermore, previously reported *trnS* and *trnM* for rice mitochondria and plastid like tRNAs, respectively [54], were additionally identified in the *O*. *minuta* mitogenome.

Searching for repeated sequences showed four direct and three inverted repeats longer than 6,050 and 70 bp, respectively (Table 4). The longest inverted and direct repeats that showed 100% identity were 112 and 19,773 bp long, respectively. Similarly, a total of 22 tandem repeats longer than 10 bp were additionally identified in the *O*. *minuta* mitogenome (Tables 4 and 5). These results were different from those previously reported for the *O*. *sativa* mitogenome, which had direct and inverted repeats of 45,584 and 946 bp, respectively [13]. Furthermore, the multipartite structure of the plant mitochondrial genome is thought to be generated through the recombination of repeated sequences; however, the involvement of these sequences in rice mtDNA is not yet clear [13]. Furthermore, the phylogenetic analysis of *O*. *minuta’s* complete mtDNA as well as 20 conserved genes with other related species revealed that it was closer to *O*. *rufipogon* than to any other related species.

## Conclusion

In this study, we reported the complete mitochondrial genome of *O*. *minuta*. The *O*. *minuta* mtDNA is composed of 515,022 bp and contained 60 known protein coding genes, two rRNA (5rRNA) and 31 tRNA genes. Genome organization and gene content is typical of the *Oryza* species and highly similar to that of *O*. *rufipogon* (89% identical at the nucleotide level). Furthermore, it shared 24 protein-coding genes, 15 tRNA genes and 1 ribosomal RNA gene with other *O*. *sativa* (*indica* and *japonica*). Similarly, the evolutionary relationship analysis with other Poaceae members revealed that the mtDNA of *O*. *minuta* is closely related to *O*. *rufipogon*. This study will improve our understanding of *O*. *minuta* (wild rice) and the evolution of the mitogenomes within the Poaceae family.

## Supporting Information

S1 Fig Heat map based on a pair-wise distance matrix of whole mitogenomes alignment as computed by Progressive Mauve. Mitochondrial genome alignments were performed using O. *minuta* as a reference genome for the other seven Poaceae members. Distance values correspond to a gradient of color steps ranging from light gray (lowest distance) to dark black (highest distance value).
(TIF)

S2 Fig Comparison of O. *minuta* mitogenome coding regions with other mitogenomes of Poaceae members using Progressive Mauve alignments. (TIF)
