# Supplementary figures and images for: Mitochondrial Genome Analysis of Wild Rice (Oryza minuta) and Its Comparison with Other Related Species
Source: PLoS One. 2016 Apr 5;11(4):e0152937. doi: 10.1371/journal.pone.0152937 (PMC4821559; doi:10.1371/journal.pone.0152937)

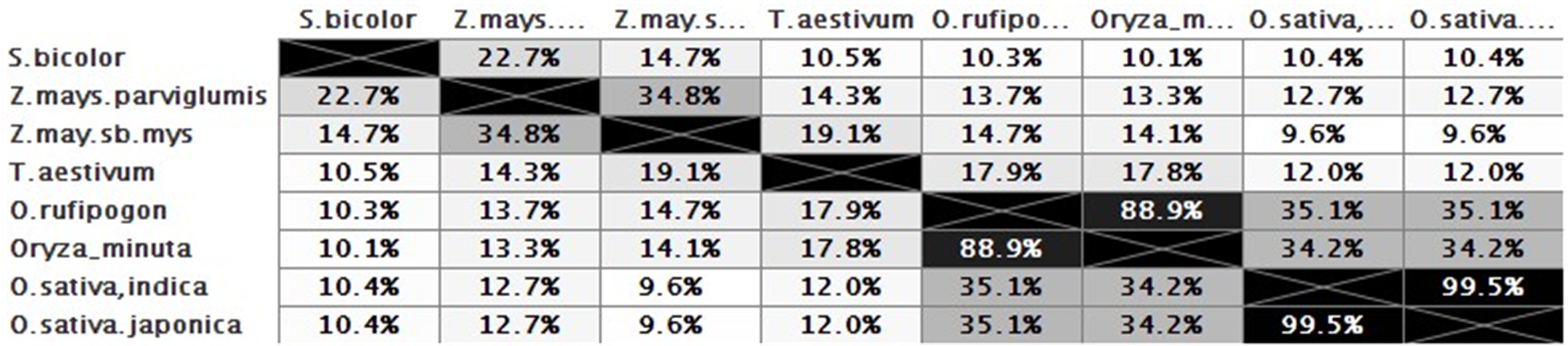

Supplement: S1 Fig — Mitochondrial genome alignments were performed using O. minuta as a reference genome for the other seven Poaceae members. Distance values correspond to a gradient of color steps ranging from light gray (lowest distance) to dark black (highest distance value). (TIF) [file pone.0152937.s001.tif]

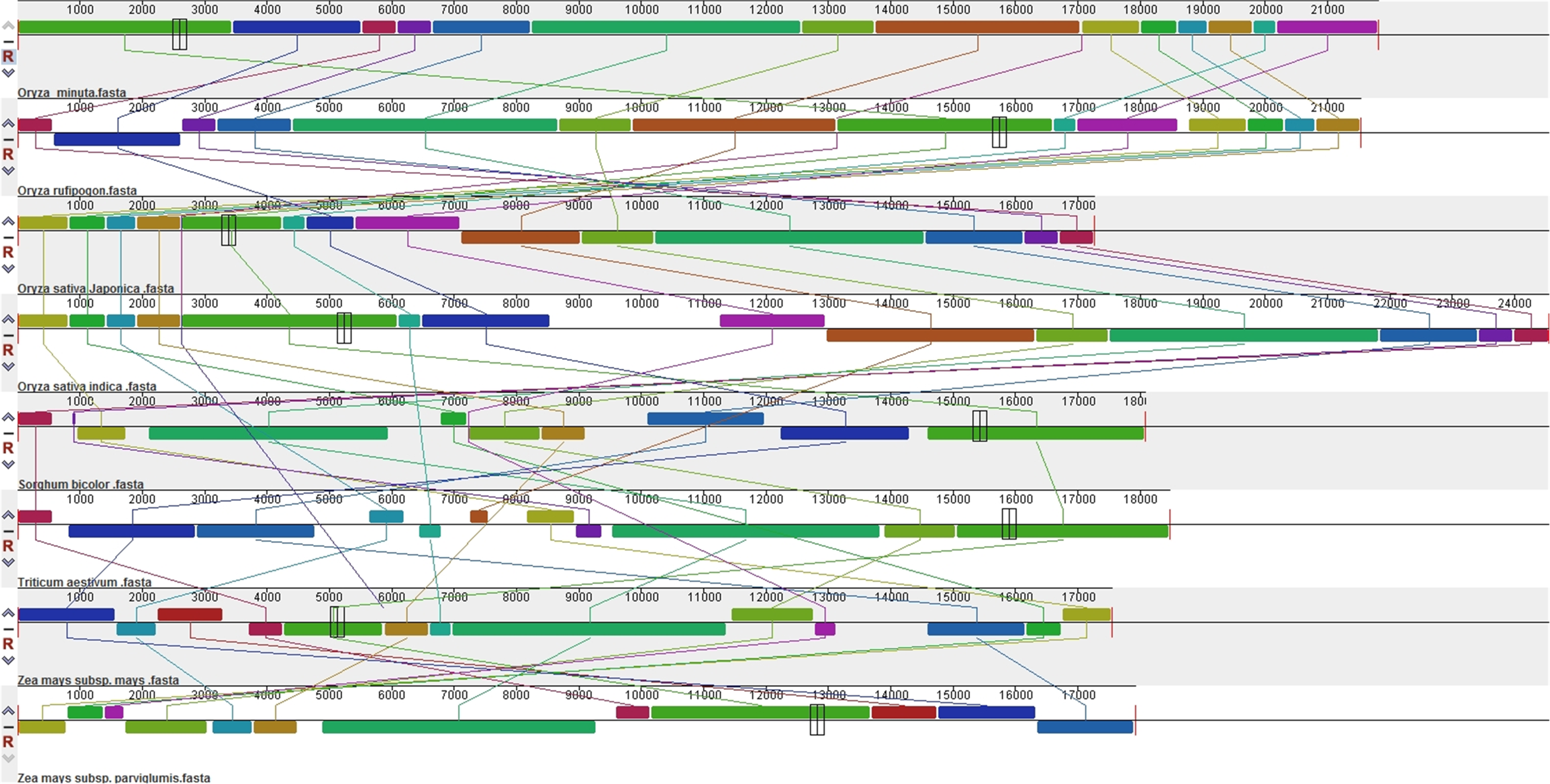

Supplement: S2 Fig — (TIF) [file pone.0152937.s002.tif]
